# Rethinking financial distress in public healthcare: evidence from Greek hospitals’ governance attributes

**DOI:** 10.3389/fpubh.2025.1690901

**Published:** 2025-09-29

**Authors:** Stefanos Karakolias

**Affiliations:** Aristotle University of Thessaloniki, Thessaloniki, Greece

**Keywords:** financial distress, Z-score, governance, public hospitals, Greece

## Abstract

**Background:**

Financial distress remains a relatively underexplored area in public healthcare, although such failures occur globally, demonstrating that maintaining public health requires strategies to ensure financial stability and the continuous operation of public healthcare organizations. This study aims to assess financial distress and its relationship with hospital-specific governance attributes by examining the case of Greek public hospitals.

**Methods:**

To achieve this aim, Altman’s Z”-score model was applied to the entire range of public hospitals. The attributes investigated included hospital size, location, specialty, and manager gender. All data were retrieved from published financial statements for 2022. The analysis employed descriptive statistics, normality tests, correlations, and non-parametric tests.

**Results:**

The findings indicate strong financial viability, reflected in high Z-scores driven by low financial leverage and ample working capital. In addition, both smaller units and women-led hospitals outperformed others in terms of Z-scores. However, heavy reliance on state subsidies, the slow collection of non-current hospital bills, and the rising levels of indebtedness suggest a financial condition substantially weaker than that implied by Z-scores or any other model based solely on financial statement data.

**Conclusion:**

Financial distress requires redefinition in the context of public entities, since their closure is not a core strategy. Existing definitions and prediction models fail to account for the support mechanisms that mask poor financial viability, effectively shifting financial distress to key stakeholders such as suppliers and the state owner.

## 1 Introduction

The health sector, including private providers, creates value by contributing to improved health outcomes. Achieving this requires a delicate balance between clinical effectiveness, patient safety, access to care, and robust accountability mechanisms, which ultimately differentiates healthcare from typical corporate structures driven primarily by shareholders’ interests. In recent years, corporate finance in healthcare organizations, particularly public units, has garnered increasing attention. A primary reason is that these organizations absorb critical public resources through government or municipal subsidies (1, 2). Additionally, their financial performance impacts private companies, for instance, through the procurement of medical supplies and drugs. Finally, even state-owned providers can fail and, in extreme cases, close down (3–5). Such failures not only affect internal stakeholders but also external stakeholders—including patients, local communities, and suppliers—who experience increased uncertainty, potentially leading to public outrage (6, 7).

Once an organization experiences poor financial performance, its liquidity, solvency, efficiency, and profitability deteriorate. Liquidity refers to the ability to meet current liabilities, such as accounts payable and other short-term obligations, using current assets, including cash, cash equivalents, and accounts receivable. The difference between current assets and current liabilities is known as “working capital.” In finance, maintaining positive working capital is essential for the smooth operation of day-to-day business activities (8).

Financial distress is the initial stage in which an organization struggles to meet its obligations to creditors on time due to persistently low or negative working capital (9, 10). Default represents the next stage, in which liabilities become overdue and creditors may legally claim the original debt plus any default interest (11). Following a prolonged period of default, organizations may lose access to additional credit, gradually deplete key resources, and experience serious disruption or interruption of core business operations (12, 13). As a result, the return on investment becomes unusually low, ultimately leading to corporate failure. In the medium term, a failed organization may declare bankruptcy and exit the market, resulting in either restructuring or dissolution/liquidation (14).

Financial distress has received limited attention in studies on public healthcare, which can be categorized into three main groups. First, some US-based studies aim to identify the factors associated with financial distress and, in turn, predict its occurrence (15–17). Second, other studies retrospectively analyze the reasons and mechanisms behind financial collapse in certain public hospitals in Europe (4, 5) and in the United States (3). Third, research examining the aftermath of financial distress primarily links it to an increased likelihood of privatization or merger (1, 18). Nevertheless, particularly in Europe, there exists a substantial body of literature on the financial performance of public hospitals (19–21), as well as on specific factors contributing to financial distress, such as indebtedness (22).

## 2 Conceptual framework and hypotheses development

### 2.1 Financial distress prediction models

It is unsurprising that academia and business continuously seek to identify early signs of financial distress. The primary objective is to propose and implement necessary restructuring actions before failure and bankruptcy become inevitable. Several prediction models have been developed for this purpose, most of which combine key financial figures to assess an organization’s financial viability.

Univariate analysis was the first method used to evaluate financial distress (23). However, due to its increasing subjectivity, it was soon succeeded by multivariate discriminant analysis (MDA). The goal of MDA is to classify entities as either “healthy” or “distressed” based on financial ratios that offer the best predictive ability. The three Z-score models (24–26) and the ZETA model (27) are the most prominent examples of MDA in the literature. An alternative approach involves calculating the probability of financial distress. Models such as the linear probability model (28) and the Lambda model (29) fall under this methodology.

It is generally accepted that models developed for commercial business activities should not be directly applied to hospitals (30). Among the models described above, the second revision of Altman’s Z-score model (25) has gained popularity in the healthcare field. This popularity stems from the fact that it is adjusted for service providers. Furthermore, recent studies on healthcare organizations have supported its reliability and accuracy (31–33).

### 2.2 Financial distress in Greek healthcare

Greek public hospitals provide a suitable context for examining financial distress. Although no bankruptcies or closures of these entities have been reported to date, evidence remains limited regarding whether they are financially healthy or at risk of distress, as previous studies have primarily conducted routine financial analyses of public hospitals (34, 35). This gap may reflect the fact that the public health system has not experienced such failures. In contrast, the local market for private hospitals and clinics has witnessed several closures, restructurings, mergers, and acquisitions, prompting researchers to assess financial distress, often using Altman’s second revised Z-score model (36). Accordingly, the first hypothesis of this study is:

*H*1. Public hospitals in Greece are not financially distressed.

### 2.3 Hospital-specific governance attributes affecting financial distress

Previous research has shown that financial distress levels may vary according to the characteristics of individual healthcare units. Differences between urban and rural hospitals have been reported, with rural hospitals appearing more vulnerable (15, 37). Size also plays a role, as smaller facilities may not benefit from economies of scale (4). Furthermore, Khullar et al. (38) found that teaching hospitals outperform other specialties in terms of liquidity. Other studies have highlighted differences based on the gender of the manager (34, 39, 40). Although these gender differences pertain to financial performance metrics rather than financial distress itself, such metrics are components of the Z-score model. Moreover, research outside the healthcare sector supports a link between manager gender and financial distress, suggesting that greater organizational diversity is associated with a lower probability of financial distress (41, 42). Based on these findings, the following hypotheses were formulated:

*H*2. The location of a hospital influences its financial distress level.

*H*3. Corporate size influences financial distress levels of Greek public hospitals.

*H*4. The specialty of a hospital influences its financial distress level.

*H*5. The gender of managers influences the financial distress levels of Greek public hospitals.

## 3 Materials and methods

### 3.1 Study design

As mentioned above, the second revision of the Z-score model has proven to be well-suited for healthcare organizations (31–33), including purely public institutions (17). Following the methodology of Ramamonjiarivelo et al. (17), this revision, known as Altman’s Z″-score (25), was used to test H1. As shown in the formula below, the overall score (Z) is the dependent variable, calculated as a linear combination of four financial ratios (independent variables), each with a different weight.


$$Z=6.56X_{1}+3.26X_{2}+6.72X_{3}+1.05X_{4}$$

where,

X_1_ = Working Capital / Total Assets, reflecting liquidity.

X_2_ = Retained Earnings / Total Assets, reflecting profitability.

X_3_ = Earnings Before Interest and Taxes (EBIT) / Total Assets, reflecting efficiency.

X_4_ = Book Value of Total Equity / Total Liabilities, reflecting solvency in the sense of financial leverage.

This model uses predetermined cutoff values to classify entities. A Z-score below 1.10 indicates financial distress, a Z-score between 1.10 and 2.60 represents a gray zone with potential risk of financial distress, and a Z-score above 2.60 signifies financial viability. However, Z-scores may lead to misclassification, as the dynamic factors contributing to financial hardship cannot be fully reflected in financial statements due to their static nature (15, 17, 34). In addition, Z-scores may misrepresent public hospitals, which operate within a state-protected environment and receive subsidies (1, 2, 43). At the same time, hospitals may become “victims” of the protection extended to other public entities—such as social insurance funds—that delay payments for hospital services (44, 45). These factors were considered when interpreting financial distress levels.

To test H2–H5, the overall Z-score (Z) and its four components were treated as dependent variables. Five independent variables were included in the analysis. For H2, a string variable was used to indicate the hospital’s location within one of the seven Regional Health Authorities (RHAs; hereafter, “RHA”). For H3, hospital size was measured as the natural logarithm of total assets and revenue (hereafter, “lnASSETS” and “lnREVENUE”), following the approach of Neves and Carolina (22) and Ziolo et al. (46). For H4, another string variable (hereafter, “SPECIALITY”) was created with five possible values: 1 for general hospitals, 2 for general hospitals with health centers, 3 for university hospitals, 4 for anticancer hospitals, and 5 for other specialties. For H5, the gender of the chief executive officer (male/female) was used as the independent variable (hereafter, “CEO”), consistent with the methodology of Karakolias and Polyzos (34).

### 3.2 Participants and data collection

This study included 90 public hospitals, representing the total population of such healthcare providers in Greece. Although more than 120 units exist, some have been unified. To calculate the Z″-score, data were obtained from the published financial statements for 2022 obtained from the published financial (47), which was the most recent year for which complete data were available. Data for testing H2-H5 were retrieved from the same source. Notably, individual financial statements were used for unified units whenever available; otherwise, consolidated financial statements were employed.

### 3.3 Data adjustments

Initially, adjustments were made to ensure the comparability of financial figures. While the vast majority of hospitals used local accounting standards, as defined by Presidential Decree 146/2003, a small group of six hospitals applied the International Public Sector Accounting Standards (IPSAS). Two main adjustments were made. First, unlike IPSAS, local accounting standards treat depreciation solely as a production cost and incorporate it into the cost of sales; the same approach was applied to hospitals using IPSAS for consistency. Second, income statements prepared under local standards include significant amounts of revenue and expenses from previous years as irregular income and expenses, respectively. These amounts were excluded from net income to align with IPSAS treatment. Subsequently, retained earnings were redefined. Since dividends are paid only by private companies, net income in public entities is fully capitalized. Therefore, in this study, retained earnings are accurately represented by net income.

### 3.4 Analysis

The research methodology was accomplished using descriptive statistics, normality tests, correlation analysis, and nonparametric tests for variables with abnormal distributions.

## 4 Results

Table 1 presents the descriptive statistics of the Z-score and its components. The mean overall score (Z) was 15.7 ± 10.8, and the median was 12.9, while the lowest score observed among the 90 hospitals was 5.5. This indicates that all examined units achieved a Z-score well above 2.60, the threshold for financial viability. This result is primarily driven by the high values of the solvency ratio (X4), despite its relatively low weight in the model: on average, hospital equity exceeded liabilities by almost 11 times, while the median value was nearly eight times. In addition to low leverage, the liquidity component (X1) also contributed to the high Z-scores. Specifically, working capital accounted for 61.7% ± 16.3% of total assets on average, with a median of 63.7%. Notably, all hospitals had positive working capital, indicating that Greek public hospitals do not appear to face liquidity issues. The results for the remaining components were mixed. Both retained earnings and EBIT, expressed as a percentage of total assets, had slightly positive mean values (X2: 1.9% ± 5.3%; X3: 0.9% ± 5.7%), though their distributions were negatively skewed. This pattern implies frequent small gains and occasional large losses, which is consistent with the not-for-profit status of these hospitals. Furthermore, the Kolmogorov–Smirnov test suggests that only X2 follows a normal distribution (*p* > 0.05); therefore, only the results of non-parametric techniques are reported below.

**Table 1:** Descriptive statistics and normality test on Z-score components.

Statistic	Z-score	X_1_	X_2_	X_3_	X_4_
Mean	15.7	61.7%	1.9%	0.9%	10.9
Median	12.9	63.7%	2.4%	1.2%	8.1
Std. Deviation	10.8	16.3%	5.3%	5.7%	10.5
Minimum	5.5	13.7%	−12.5%	−16.7%	1.6
Maximum	80.7	86.1%	21.0%	17.4%	74.3
Range	75.2	72.5%	33.5%	34.1%	72.7
Interquartile Range	6.9	17.7%	6.2%	5.6%	7.2
Skewness	3.5	−0.9	0.0	−0.4	3.7
Kurtosis	16.7	0.6	1.6	1.6	17.4
K-S statistic^a^	0.115	0.083	0.117	0.241	0.223
K-S Sig.^a^	0.005	0.173	0.004	0.000	0.000

a Kolmogorov–Smirnov test.

Table 2 reports the correlations between numerical variables. As expected, the two size measures were positively and almost perfectly correlated (*ρ* = 0.967, *p* < 0.05), indicating that higher asset values correspond to higher revenue, and vice versa. In addition, a weak but significant negative correlation was observed between Z-scores and size, measured by the natural logarithm of revenue (*ρ* = −0.266, *p* < 0.05). This suggests a disadvantage for larger hospitals in terms of financial viability, primarily because larger hospitals operate with higher leverage (X4: *ρ* = −0.356, *p* < 0.05), which offsets their superior profitability and efficiency (X2: ρ = 0.356, *p* < 0.05; X3: ρ = 0.390, *p* < 0.05).

**Table 2:** Correlations between Z-score components and hospital size.

Variable	Spearman’s rho	LnASSETS	LnREVENUE	X_1_	X_2_	X_3_	X_4_	Z-score
LnASSETS	Coefficient	1.000	0.967^**^	0.143	0.321^**^	0.354^**^	−0.240^*^	−0.160
	Sig. (2-tailed)		0.000	0.178	0.002	0.001	0.023	0.131
LnREVENUE	Coefficient	0.967^**^	1.000	0.170	0.356^**^	0.390^**^	−0.356^**^	−0.266^*^
	Sig. (2-tailed)	0.000		0.109	0.001	0.000	0.001	0.011

**Correlation is significant at the 0.01 level (2-tailed). *Correlation is significant at the 0.05 level (2-tailed).

Regarding gender, Table 3 indicates that female-led hospitals achieved significantly higher Z-scores, suggesting a lower risk of financial distress (*p* < 0.05). This advantage is mainly attributable to lower leverage (X4), though the statistical significance for this component is marginal (*p* = 0.06).

**Table 3:** Z-score components by CEO’s gender.

Dependent variable	Grouping variable: CEO	N	Mean rank	Mann–Whitney U	Wilcoxon W	Z	Asymp. Sig. (2-tailed)
X_1_	Male	69	45.32	712	3,127	−0.119	0.905
	Female	21	46.10				
X_2_	Male	69	45.61	717	948	−0.072	0.943
	Female	21	45.14				
X_3_	Male	69	45.75	707	938	−0.167	0.867
	Female	21	44.67				
X_4_	Male	69	42.64	527	2,942	−1.884	0.060
	Female	21	54.90				
Z-score	Male	69	42.41	511	2,926	−2.037	0.042
	Female	21	55.67				

Results for other hospital-specific attributes were less conclusive. Table 4 shows that hospital location did not significantly affect overall Z-scores (*p* > 0.05). However, significant differences were observed for components X1-X3 (*p* < 0.05). Specifically, hospitals in northern Greece (3rd and 4th RHAs) exhibited higher working capital (X1). These hospitals, along with those in Crete (7th RHA), were significantly more profitable (X2) and more efficient (X3). Similarly, Table 5 suggests that hospital specialty had no significant impact on Z-scores or their components (*p* > 0.05). Nevertheless, at a slightly higher significance threshold, anticancer hospitals were more efficient (X3) than hospitals of other specialties (*p* = 0.06).

**Table 4:** Z-score components by hospital location.

Dependent variable	Grouping variable: RHA	N	Mean Rank	Kruskal-Wallis H	Asymp. Sig.
X_1_	1st	12	35.58	25.597	0.000
	2nd	16	33.50		
	3rd	11	67.36		
	4th	13	65.85		
	5th	10	53.10		
	6th	22	34.59		
	7th	6	40.50		
X_2_	1st	12	42.67	15.213	0.019
	2nd	16	34.63		
	3rd	11	65.73		
	4th	13	54.62		
	5th	10	42.10		
	6th	22	37.18		
	7th	6	59.50		
X_3_	1st	12	38.00	22.782	0.001
	2nd	16	35.63		
	3rd	11	67.45		
	4th	13	62.62		
	5th	10	37.30		
	6th	22	35.41		
	7th	6	60.17		
X_4_	1st	12	41.08	5.997	0.424
	2nd	16	39.06		
	3rd	11	39.09		
	4th	13	51.15		
	5th	10	38.30		
	6th	22	53.41		
	7th	6	54.00		
Z-score	1st	12	41.25	6.656	0.354
	2nd	16	34.75		
	3rd	11	47.18		
	4th	13	54.77		
	5th	10	38.80		
	6th	22	50.09		
	7th	6	53.83		

**Table 5:** Z-score components by hospital specialty.

Dependent variable	Grouping variable: specialty^†^	N	Mean rank	Kruskal-Wallis H	Asymp. Sig.
X_1_	1	68	48.93	6.528	0.163
	2	7	30.57		
	3	7	42.29		
	4	2	47.50		
	5	6	27.17		
X_2_	1	68	47.22	7.630	0.106
	2	7	36.57		
	3	7	45.71		
	4	2	77.50		
	5	6	25.50		
X_3_	1	68	45.87	9.061	0.060
	2	7	33.43		
	3	7	54.86		
	4	2	86.00		
	5	6	31.00		
X_4_	1	68	45.65	2.834	0.586
	2	7	50.86		
	3	7	32.43		
	4	2	40.50		
	5	6	54.50		
Z-score	1	68	46.25	2.275	0.685
	2	7	47.71		
	3	7	31.57		
	4	2	48.50		
	5	6	49.67		

† 1: general hospital, 2: general hospital-health center, 3: university hospital, 4: anti-cancer hospital, 5: other.

## 5 Discussion

### 5.1 Hypotheses overview and policy implications

The empirical findings presented in the previous section provide evidence for evaluating the research hypotheses.

H1 is accepted, as no hospital was classified as financially distressed (Z < 1.10) or at risk of financial distress (1.10 ≤ Z ≤ 2.60) according to Altman’s Z″-score. However, this result warrants a more in-depth analysis, as discussed below.

H2 is partially supported. While hospital location appears to influence three of the four Z-score components, it does not result in statistically significant differences in overall Z-scores. Hospitals situated in predominantly rural areas outperform their counterparts in terms of liquidity, profitability, and efficiency. Here, “predominantly” indicates that these high-performing hospitals are mainly located in the 3rd, 4th, and 7th RHAs, which include mostly rural regions but also encompass urban areas such as Thessaloniki—the co-capital of Greece—and major cities in Crete. Although these findings are not directly comparable to those of Malone et al. (37) and Holmes et al. (15), they do not support the claim that rural hospitals are particularly vulnerable to financial distress. Nevertheless, the variation in financial performance across geographic areas suggests that policymakers and central administration should develop mechanisms to monitor and address these discrepancies.

H3 is supported, as smaller hospitals exhibited significantly higher Z-scores. This outcome reflects lower leverage, despite lower profitability and efficiency, compared to larger hospitals. This finding contrasts with those of Mosciaro et al. (8) and Langabeer et al. (48), who reported that smaller facilities are generally in poorer financial condition. These results suggest that policymakers should tailor financial oversight and support based on hospital size. Larger hospitals, with higher leverage and lower Z-scores, may require targeted interventions to manage debt and enhance financial stability, while best practices from smaller, more resilient hospitals could inform efficiency and liquidity improvements.

H4 is rejected, as hospital specialty had no significant impact on either the Z-score or its components. This contradicts Khullar et al. (38), who suggested that hospitals with higher liquidity are more likely to be university hospitals.

H5 is supported. Hospitals with female CEOs achieved higher Z-scores, primarily driven by better solvency ratios. This result aligns with prior studies indicating that women in C-suite positions contribute to reducing organizational leverage, either immediately (49) or over the short term (34), consistent with observed tendencies of female leaders to adopt less risky and less aggressive financial strategies (50). These findings suggest that promoting gender diversity in hospital leadership could enhance financial stability. Policymakers and healthcare administrators may consider initiatives to support the recruitment, retention, and advancement of women into executive positions. Furthermore, leadership training programs could integrate financial risk management practices demonstrated by female executives to improve overall organizational solvency.

### 5.2 Insights into Z-score components

As noted above, the surprising finding that all examined units were classified as financially viable warrants further investigation. First, the Z-score composition highlights the moderating role of leverage (X4) and working capital (X1). Specifically, 11.5 out of the mean Z-score of 15.7 are contributed by X4, while X1 contributes an additional 4.1 points. Consequently, the Z-score effectively becomes a linear function of these two financial ratios in this sector, underscoring the need for a closer examination of X1 and X4.

X4 reflects the equity-to-liabilities ratio. The equity of Greek public hospitals comprises initial capital, donations, state subsidies for investment purposes, and retained earnings. In 2022, the sector’s cumulative net income reached €336 million but relied on state subsidies of approximately €1.9 billion, intended to cover permanent staff payroll and other operational expenses. According to Karakolias and Polyzos (34), only three hospitals could self-finance their operations without subsidies in 2022. This indicates that a substantial portion of equity consists of external financing rather than internally generated capital. Moreover, these organizations do not pay dividends, so net income is fully retained. Long-term liabilities are virtually absent because fixed assets are financed solely through state subsidies (for investment) and donations, and short-term bank loans are not used to support working capital. As a result, liabilities primarily comprise short-term obligations to suppliers and tax or social insurance contributions. Another peculiarity arises from the “clawback” mechanism: suppliers repay the portion of their sales that exceeds the state’s global budget (51), making them simultaneously debtors and creditors. Claims from hospitals are often offset by clawback. In summary, Greek public hospitals exhibit two unique governance features: equity largely contributed by external sources and de facto limited and controlled debt. Accordingly, X4 tends to understate financial leverage, leading to inflated Z-scores.

Working capital, a key component of X1, depends on current assets and liabilities. Inventory, accounts receivable (A/R), cash, and cash equivalents constitute the bulk of current assets. In 2022, A/R accounted for 93.5% of current assets, while cash and cash equivalents comprised only 3.5%. This indicates that liquidity is largely tied to receivables. Greek public hospitals are primarily financed under the Bismarck model, meaning services are provided on credit to the unified sickness fund (EOPYY) and a few other statutory insurance funds. A/R therefore represents claims from these entities. A recent study examining activity ratios in 2022—ratios not incorporated in Altman’s Z″-score—revealed a substantial gap between cash inflows and outflows: claims are collected after an average of 1,485 days (“accounts receivable days”), while current liabilities are settled in 93 days (“accounts payable days”) (34). This raises the question of how “current” an asset is if it is converted to cash after 4 years. IPSAS guidelines classify assets collected after 1 year as non-current. Moreover, X1 overstates actual liquidity by ignoring limited liquid assets, which inflates Z-scores and exaggerates the hospitals’ ability to absorb large financial shocks (38).

As noted, Greek public hospitals face delayed payments from social insurance schemes, a problem also observed in other health systems (44). This typically results in deferred payments to suppliers to bridge the gap between cash inflows and outflows. Larger hospitals often exploit their market position to dictate payment terms (52), so late payments primarily affect smaller local suppliers, undermining competition. Larger suppliers may charge interest or refuse to supply, worsening liquidity in a vicious cycle (53). Portugal illustrates this issue: public hospitals there had an average accounts payable days ratio of 231 (21), and researchers continue to investigate the sources of such indebtedness (22). By comparison, Greek public hospitals settle their current liabilities much faster (average 93 days), though roughly half exceed 90 days—the threshold for overdue liabilities. This pattern is comparable to the Polish case, where 40% of public hospitals had overdue liabilities a few years ago (19), with little improvement to date (54).

### 5.3 Limitations and directions for future research

The main limitation of this study is that it covers a single fiscal year (2022) and, therefore, cannot capture trends over time. This limitation is due to significant lags in financial reporting, as most hospitals have not yet published their financial statements for 2023 and 2024. Additionally, earlier years prior to 2022 were excluded because the effects of COVID-19 could not be isolated in the data.

It should also be noted that the overdue liabilities of Greek public hospitals totaled €344 million at the end of 2019, increased to €907 million by the end of 2022, and reached €1,164 million by the end of 2024 (55). This represents a more than threefold increase since 2019 and a 28% rise since 2022. In practice, the hospitals examined not only benefit from clawback offsets, as discussed earlier, but also manage their debt by delaying payments to suppliers. These delays tend to expand over time, in line with EOPYY’s payment schedules. Consequently, forthcoming financial statements for 2023 and 2024 could show even higher Z-scores if debt grows more slowly than receivables. Nevertheless, both trends point to a more disrupted trade cycle, which could have significant implications for hospital operations.

Accordingly, future research should aim to validate Z-scores over time and refine them to capture subsidy dependence, delayed cash inflows, and other structural vulnerabilities specific to public healthcare organizations.

## 6 Conclusion

In conclusion, the application of Altman’s Z″-score to Greek public hospitals revealed neither financial distress nor any imminent risk in the narrow sense. Indeed, there is evidence of superior financial viability for smaller hospitals and those led by female executives. However, when examining factors beyond Z-scores, several characteristics were identified that have previously been associated with severely financially distressed public organizations (5, 43, 46): (i) substantial ongoing state subsidies used to finance fixed assets and operational expenses; (ii) limited cash liquidity coupled with stagnant claims from health insurance funds; (iii) rising indebtedness; and (iv) the systemic ability to shift budget and cash imbalances to suppliers through clawback mechanisms and delayed (often overdue) payments.

This finding does not constitute a critique of Altman’s model or the fundamental definitions of financial distress. Rather, it suggests that any definition or model based solely on financial statement data is likely to overstate financial health, precisely because the financial statements of such public entities are prepared after the state—acting as owner—has absorbed major financial deficits or allowed them to be passed on to creditors.

In this way, the governance of public hospitals resembles a continuous state-administered bailout. From a policy perspective, this is partly justified given the hospitals’ central role in health systems. However, from a strict management standpoint, it obscures the true financial condition of these organizations, so that financial distress would only be uncovered retrospectively, even when all warning signs are present (3–5).

Therefore, this study paves the way for redefining financial distress in public healthcare organizations, for which bankruptcy is not an option. A key notion is that such organizations may be considered inherently financially distressed once they become entirely dependent on government funding in perpetuity, while simultaneously imposing financial pressure on their suppliers. This implies that such supporting mechanisms should be transparently disclosed in financial reports.

## Data Availability

The original contributions presented in the study are included in the article/supplementary material, further inquiries can be directed to the corresponding author.
